# Towards Elimination of Sexually Transmitted Infections: Research Developments and Vaccine Uptake

**DOI:** 10.1002/jia2.70173

**Published:** 2026-07-24

**Authors:** Jeanne Marrazzo, Nelly R. Mugo, Yves Levy

**Affiliations:** ^1^ Infectious Diseases Society of America Arlington Virginia USA; ^2^ Department of Global Health University of Washington Seattle Washington USA; ^3^ Center for Clinical Research Kenya Medical Research Institute Nairobi Kenya; ^4^ Vaccine Research Institute Inserm U955, Université Paris‐Est Créteil, Faculté de Médicine Créteil France

At first glance, we should be living in a golden age for the potential of immunization against sexually transmitted infections (STIs). Vaccination against oncogenic human papillomavirus (HPV) has demonstrated that we can eliminate cervical cancer [1]. We have developed new platforms that can rapidly adapt to emerging threats, notably mRNA technology, which underwent proof‐of‐concept trials during the COVID‐19 pandemic [2]. Monoclonal antibodies have been rapidly developed and wielded to fight deadly viruses, like Ebola and Marburg, and played a pivotal role in prevention and therapy for SARS‐CoV‐2. Cutting‐edge approaches to immunogen design have harnessed the power of increasingly sophisticated structural biology techniques to target hidden immunogenic sites, and artificial intelligence is helping to direct approaches to subvert ever‐evolving pathogenic defences.

Unfortunately, the STI field has not yet benefited fully from these advances despite the critical need for STI‐focused prevention innovations. Western countries are experiencing some of the highest incidence rates ever of *Neisseria gonorrhoeae* (gonorrhoea)*, Treponema pallidum* (syphilis) and *Chlamydia trachomatis* (chlamydia) since these epidemics were first tracked. Congenital syphilis cases have reached record numbers in several countries, undermining the successful elimination efforts of over 20 countries [3]. These rising secular trends have been attributed to condomless sex in high socio‐demographic index regions and increased numbers of people in the age bands with highest disease burden, among other factors.

Nearly one‐third of sexually acquired HIV cases are attributable to genital infection with herpes simplex virus type‐2 (HSV‐2) [4]. With concern for sustaining pre‐PEPFAR levels of antiretroviral‐mediated viral suppression and pre‐exposure prophylaxis in an era of constrained resources, this proportion may increase. We continue to encounter outbreaks of “new” STIs, notably Mpox, dermatophytosis caused by *Trichophyton mentagrophytes* Genotype VII and folliculitis caused by *Klebsiella aerogenes* [5]. The role of *Mycoplasma genitalium* in potentiating HIV acquisition or transmission remains unclear, but this pathogen can be extremely challenging to treat and likely increase risk of female upper reproductive tract inflammation.

In this supplement of the *JIAS*, we address several of these challenges with perspectives from investigators across the globe. Perhaps the most broadly relevant is the paper by Firth and Hodgson that discusses the role of stigma in confounding any intervention aimed at controlling STIs, especially vaccines. For STI vaccines to have adequate uptake, one of two conditions must occur. Either people are vaccinated prior to sexual debut—the ideal approach taken with HPV vaccination at the population level—or they must elect to be vaccinated, a decision that is largely based on perception of STI risk. Acknowledging risk of STI acquisition can invoke shame, self‐judgement and stigma—all of which can greatly impact the likelihood of vaccine acceptance. These authors frame their discussion around three key STIs for which vaccination is or will be critical to control: HIV, which invoked intense moralization; HPV, for which the implementation approach has been “sanitized”; and Mpox, which illustrates the power of marginalization of traditionally stigmatized groups (in this case, men who have sex with men) [6].

The STI field has never suffered from a lack of human investment, but unfortunately, capital investment has not been commensurate. Funding for basic and translational science in HIV has long dwarfed the financial and operational investment in STI control, despite the issuance of several roadmaps to prioritize new vaccines that might conquer STIs. In this context, Peters et al. review how STI vaccine development has proceeded in the last decade. Using an example from HPV vaccination rollout, the authors recommend a combined strategy that includes strong community education to raise awareness, addressing misconceptions and vaccine mistrust with active engagement of adolescents, and instituting systematic prompts for healthcare providers [7].

Despite these limitations, there are some bright spots. The recognition that immunization against *N. meningitidis* conferred partial benefit against acquisition of gonorrhoea sparked several clinical trials to rigorously examine this effect—several of which are ongoing, the rationale for which is explored in the paper by Gottlieb et al. [8]. Equally important, these findings energized the field to pursue a detailed definition of what underlies this apparent “crossover” immunoprotective effect between *N. gonorrhoeae* and *N. meningitidis*—an approach that is yielding new immunogens and a new agenda for future study—as detailed by Seib and Grulich [9]. The HPV vaccine continues to exert global impact; as Doyle et al. discuss, mathematical modelling demonstrates that even catch‐up immunization has the potential to lead to major reductions in the incidence of oncogenic HPV acquisition and cervical cancer [10].

Despite these promising insights, barriers remain; Peter et al. detail structural impediments to implementing widescale HPV vaccination in Nigeria and ways to overcome them. Even with the positive effect of the interventions they describe, uptake of HPV immunization remained very low among the adolescents studied [11]. Finally, implementation of doxycycline post‐exposure prophylaxis is demonstrating community‐level impact in several regions that have made it available for several years, particularly on the incidence of syphilis and chlamydia. While this is encouraging, concerns about concomitant pressure on the evolution of antibiotic resistance, the absence of protection against gonorrhoea acquisition and the likelihood of widespread uptake among some populations—particularly cisgender women—raise concerns about the durability of this approach over the longer term. It is likely that immunization approaches, especially for syphilis and gonorrhoea, will be needed to fully effect control in the coming decade [12, 13, 14].

Sexual minority and other vulnerable populations—particularly those at risk of HIV acquisition—experience some of the biggest challenges to attaining sexual health, and safe and easy access to vaccination is one of these, as addressed by the articles by Rapozo et al. and Silva et al. [15, 16]. Even when safe and effective vaccines are available, immunologic durability and uptake can be challenging, as discussed by Palmore and Paulino‐Ramirez et al., respectively, for the currently available Mpox vaccines [17, 18].

The landscape of vaccines in general has been fraught with threats to funding, increasing scepticism, loss of infrastructure for delivery and misinformation. Vaccines aimed at STIs face the additional challenges outlined above: lower priority for funding, shame and stigma around sexual health, and the fact that the most affected populations are often marginalized. Yet, the cost of inaction is substantial. Globally, there is an estimate of over 1 million incident cases of STIs, with 8.22 million disability‐adjusted life years associated with the five most common STIs and 7.26 million years of life lost due to congenital syphilis [19]. STIs increase the risk for infertility, preterm births, stillbirths and other pregnancy‐specific complications, in addition to HIV acquisition and the intercurrent social‐economic sequelae. Effective STI vaccines could mitigate the challenges posed by the lack of appropriate diagnostic tools and shortcomings in health systems found in low‐resource settings, where individuals carry disproportionate burden of STI disease sequelae. Addressing these challenges means that we must confront them and articulate a path forward, as this supplement aims to do.

## Author Contributions

JM drafted the editorial with inputs from NRM and YL. All authors reviewed and approved the final version.

## Funding

This supplement was organized by IAS—the International AIDS Society—with financial support from ANRS | Emerging Infectious Diseases.

## Conflicts of Interest

The authors declare no conflicts of interest.

## Data Availability

Data sharing not applicable to this article as no datasets were generated or analysed during the current study.
